# Acknowledgement to Reviewers of *Nanomaterials* in 2015

**DOI:** 10.3390/nano6010023

**Published:** 2016-01-21

**Authors:** Nanomaterials Editorial Office

**Affiliations:** MDPI AG, Klybeckstrasse 64, CH-4057 Basel, Switzerland; nanomaterials@mdpi.com

The editors of *Nanomaterials* would like to express their sincere gratitude to the following reviewers for assessing manuscripts in 2015.

We greatly appreciate the contribution of expert reviewers, which is crucial to the journal’s editorial decision-making process. Several steps have been taken in 2015 to thank and acknowledge reviewers. Good, timely reviews are rewarded with a discount off their next MDPI publication. By creating an account on the submission system, reviewers can access details of their past reviews, see the comments of other reviewers, and download a letter of acknowledgement for their records. In addition, MDPI has launched a collaboration with Publons, a website that seeks to publicly acknowledge reviewers on a per journal basis. This is all done, of course, within the constraints of reviewer confidentiality. Feedback from reviewers shows that most see their task as a voluntary and mostly unseen work in service to the scientific community. We are grateful to our reviewers for the contribution they make.

Abu-Lebdeh, Yaser
Addleman, Raymond S.
Ajji, Abdellah
Akram, Muhammad S.
Aksimentiev, Aleksei
Albrecht, Eike
Alexis, Frank
Alonso-Vante, Nicolas
An, Linan
Anastasiadis, Spiros H.
Anderson, Anne J.
Angel, Brad
Ariga, Katsuhiko
Armentano, I.
Arya, Sunil Kumar
Asuri, Prashanth
Atkuri, Hari
Bach, Horacio
Balbuena, Perla
Barbillon, Grégory
Başağaoğlu, Hakan
Batley, Graeme
Ben-Yoav, Hadar
Bernard, Samuel
Bernède, J. C.
Berrier, Elise
Bertsch, Paul
Bhargava, Suresh
Biernat, Jan
Billaud, Juliette
Birkett, Martin
Bisesi, Joseph
Bishop, Alistair
Boldt, Klaus
Bordia, Rajendra
Bouyer, Frederic
Brownson, Dale A. C.
Cabot, Andreu
Capiglia, Claudio
Carter, Kimberly
Casey, Alan
Causin, Valerio
Celano, Umberto
Cellesi, Francesco
Celzard, Alain
Cevenini, Luca
Chan, Yong Shin
Chang, Ting-Chang
Chen, Chiing-Chang
Chen, Chuh-Yung
Chen, George
Chen, Hong
Chen, Huaiqiong
Chen, Jun
Chen, Shi-Jie
Chen, Yu
Chi, Qijin
Chiang, Ming-Hsi
Chieruzzi, Manila
Chiolerio, Alessandro
Chowdhury, Sanjib
Chrysovalado, Deimede
Chu, Dafeng
Chuang, Peng
Chubar, Natalia
Ciardelli, Francesco
Clift, Martin
Coffinier, Yannick
Coll, Mariona
Colomer, M. T.
Compton, Richard
Cordoba-Diaz, D.
Correia, Ilídio J.
Cortajarena, Aitziber L.
Creager, Stephen E.
Cremar, Lee Daniel
Critchley, Kevin
Cui, Hui-Wang
Cummins, Gerard
Dal Negro, Luca
Daniele, Salvatore
Davis, Chelsea
de la Hoz, Antonio
De Rosis, Alessandro
Dekaban, Gregory A.
Del Gaudio, Costantino
Delogu, Francesco
DeRouchey, Jason
Diaz, Edgar
Dickert, Franz L.
Ding, Baoquan
Ding, Daowei
Donald, William A.
Doña-Rodríguez, José M.
Donnelly, Ryan F.
Doris, Eric
Dorman, James
Doye, Jonathan
Dunne, Nicholas
Eigler, Siegfried
Falcón, M.A.
Falletta, Ermelinda
Fan, Donglei
Fang, Jian
Fauzi, Iliana
Feng, Ying
Ferré-Borrull, Josep
Finch, Caleb E.
Flores, Octavio
Francia, Carlotta
Fridman, Gregory
Frippiat, Christopher
Fröhlich, Eleonore
Fudouzi, Hiroshi
Fujita, T.
Gao, Wei
Gaponenko, Sergey
Garbuzenko, Olga
García-Martinez, Joaquín
Gardelis, Spiros
Garrett, David J
Gartia, Manas Ranjan
Gary-Bobo, Magali
Gattacceca, Florence
Gatti, Antonietta
Ghafainazari, Ali
Giannazzo, F.
Gil, A.
Gil, Eduardo Pinilla
Glass, Jeffrey T.
Goldoni, Matteo
Gómez-Acebo, Tomás
González, Blanca
Gopalakrishnan, Sai G.
Graczyk-Zajac, M.
Guerrero, Miguel
Gurikov, Pavel
Haberko, Krzystof
Hak, Sjoerd
Hamilton, James P.
Han, Zhaojun
Harvey, Stephen C.
Hayakawa, Tohru
HAYASHI, Masahiko
He, Jie
He, Jun
He, Ping
He, Xiaojia
Henry, Ted
Herrero, María Antonia
Hippler, Rainer
Hirsch, Thomas
Hong, Liang
Honma, Itaru
Hou, Chong
Hu, Anming
Hu, Shan
Huang, Chih-Ching
Huang, Huang-Chiao
Hung, Wei Hsuan
Hwang, Young Kyu
Hyodo, Fuminori
Ide, Keisuke
Ilg, Patrick
Iniesta, Jesús
Ionescu, Emanuel
Ishigaki, Takamasa
Islam, Syed Kamrul
Isoda, Katsuhiro
Ivey, Douglas
Jang, Kiwan
Jasinski, Jacek B.
Jenny, Titus A.
Jessel, Nadia
Jiang, Junhua
Jobic, Stéphane
Joglekar, Madhura
Jones, Harlan P.
Kadib, Abdelkrim El
Kako, Tetsuya
Kalantar-Zadeha, K.
Kalinowski, Danuta
Kamada, Kai
Kamps, Jan A. A. M.
Kanamura, Kiyoshi
Karasali, Helen
Karthaus, Olaf
Kasiotis, Konstantinos M.
Kast, Richard
Kavishwar, Amol
Kawanago, Takamasa
Kelder, Erik M.
Kermanizadeh, Ali
Khan, Mujibur R.
Khodadoust, Amid P.
Khodaparast, G. A.
Khosla, Ajit
Kichambare, Padmakar
Kidoaki, Satoru
Kim, Hak Yong
Kim, Il Tae
Kim, Jaheon
Kim, Ji-Hee
Kim, Jong Ho
Kim, Jung Ho
Kim, Sang-Jae
Kim, Sung-Kon
Kim, Yoong Ahm
King, Sean
Kitagishi, Hiroaki
Klontzas, Michael
Ko, Young Tag
Kolesnichenko, Vladimir
Korampally, V.
Kowalska, Ewa
Kraehnert, Ralph
Krause, Beate
Krauss, Thomas F.
Krug, Harald F.
Kuang, Qin
Kucuk, Israfil
Kumai, Y.
Kuriakose, Aneetta
Kuzuya, Akinori
Lai, Yi Sheng
Lakard, Boris
Lalatonne, Yoann
Lan, Zhiyuan
Landy, David
Langer, Jerzy J
Lapotko, Dmitri O.
Larue, Camille
Lee, Chang-Seop
Lee, Dongwon
Lee, HaeYeon
Lee, Hsien-Ming
Lee, Su Seong
Lee, Sunwoo
Lee, Tzong-Shyuan
Lefebvre, David E.
Lefèvre, Grégory
Lemarchand, Karine
Leong, David Tai
Leporatti, Stefano
Levchenko, Igor
Li, Jing
Li, Pei-Wen
Li, Robert K. Y.
Li, Yali
Li, Yongsheng
Li, Zhanjun
Liang, Xishuang
Liao, Yougui
Lim, Chang-Sung
Lim, Soonho
Lin, Chih-Ming
Lin, Hsiu-Li
Lin, Tzy-Rong
Ling, Bo
Ling, Maurice Ht
Liu, Bin
Liu, Chee-Wee
Liu, Dongsheng
Liu, Fang
Liu, Gao
Liu, Jun
Liu, Min
Liu, Xiaogang
Lohmüller, Theobald
Long, Yi
Longoni, Gianluca
Lou, Xiangwen
Lu, Yang
Luo, He-Kuan
Lyalin, Andrey
Lykakis, Ioannis
Ma, Wanhong
Maeda, Isamu
Maiyalagan, T.
Male, David K.
Malik, Muhammad Arif
Malucelli, Giulio
Manthina, Venkata
Marchesan, Silvia
Marinas, A.
Martinez-Manez, Ramon
Maskos, Michael
Matten, Sharlene R.
McGoron, Anthony J.
Mehring, Michael
Melin, Frederic
Mendoza, Miller
Mera, Gabriela
Merino, Sonia
Mijangos, Carmen
Milanese, M.
Minakshi, Manickam
Minero, Claudio
Ming, Goh Xiao
Minuesa, Eduardo Solano
Miomandre, Fabien
Miroshnichenk, Andrey
Mishra, Yogendra K.
Mitchell, John
Mitra, Somenath
Miyoshi, Hirokazu
Moghaddam, Minoo
Mokaya, Robert
Monguzzi, Angelo
Moriwaki, Hiroshi
Morris, Michael
Mukherjee, S.
Muneshwar, Triratna
Nagaraj, Vinay J.
Naik, Rajesh
Nair, Sankar
Nakagawa, Yoshinao
Nakano, Hideyuki
Nakayama, Masaharu
Nalayanda, Divya
Narciso, Javier
Nganga, Sara
Ni, Jiangfeng
Nicholls, Ian
Niidome, Takuro
Niikura, Kenichi
Nishihara, Hirotomo
Nishizawa, Matsuhiko
Nonoguchi, Yoshiyuki
Nowicka, Anna Maria
Oh, Si Hyoung
Oh, Won-Chun
Otero, T. F.
Ouldridge, Thomas
Ozkan, Mihrimah
Paci, M.
Page, Alister
Pagura, Cesare
Pang, Xinchang
Paolone, Annalisa
Papoulis, Dimitrios
Parac-Vogt, Tatjana N.
Park, Hyung Gyu
Park, O Ok
Park, Wook
Park, Yong Il
Pasquato, Lucia
Pastoriza-Santos, Isabel
Patterson, Jennifer
Paulmurugan, Ramasamy
Peigney, A.
Peijnenburg, Willie
Peng, Zhiwei
Pérez García, Manuel
Pérez-García, Lluïsa
Petersen, Elijah
Petty, Jeffrey T.
Pfleging, Wilhelm
Philipose, U.
Piao, Yuanzhe
Pinto, Artur
Piraino, Francesco
Pita, Marcos
Pons, Ramon
Pope, Michael
Poupot, Remy
Prati, Laura
Prawer, Steven
Prida, V. M.
Pu, Ying-Chih
Rädler, Joachim O.
Rahman, Masudur
Raj, Rishi
Venkateswara Rao, C.
Ratnakumar, B. V.
Rawat, Rajdeep
Rebollar, Esther
Reddy, M. V.
Rekas, Mieczyslaw
Richard, Cyrille
Robertson, John
Roh, Kwang Chul
Ronkainen, Niina J.
Rose, Marcus
Rotello, Vincent M
Ryu, Sung Hun
Ryu, Won-Hee
Sakaebe, Hikari
Sakata, Masayo
Saleem, Imran
Salieri, Beatrice
Salm, Cora
Samaroo, Diana
Sanaty-Zadeh, A.
Santos, Lídia
Scaggiante, Bruna
Scheicher, Ralph H.
Schiraldi, David
Schirhagl, Romana
Segawa, Hiroyo
Seguini, Gabriele
Semboshi, Satoshi
Shao, Yong
Shaposhnik, Zory
Sharifi, Tiva
Shimoi, Norihiro
Sicard, Lorette
Singh, Gurpreet
Smeltzer, Mark S.
Song, Changsik
Song, Hyun-Kon
Song, Jinhui
Song, Kenan
Song, Min Kyu
Sooter, Letha J.
Sorarù, Gian Domenico
Sotiropoulos, S.
Sourice, J.
Srivastava, Gyaneshwar
Strelniker, Yakov M.
Stroeve, Pieter
Strunk, Jennifer
Suh, Yung Doug
Sun, Baoyun
Sun, Lu
Taboada, Pablo
Takeguchi, Tatsuya
Takeno, Hiroyuki
Tanaka, Katsufumi
Tao, Hu
Tavangarian, Fariborz
Tei, Lorenzo
Terashima, Takaya
Teychene, Benoit
Thomas, Joan
Thompson, Van P.
Thorley, Andrew J.
Thota, Sammaiah
Toney, Mike
Torigoe, Kanjiro
Toshihiko, Fujimori
Trif, Monica
Trojanowicz, Bogusz
Tsarev, Andrei
Tse, Brian
Tsuyumoto, Isao
Upadhyay, Arun Kumar
Vadlapudi, Aswani Dutt
Vaidya, Bhuvaneshwar
Valiyaveettil, Suresh
Valov, I.
Vázquez, Ester
Venditti, Iole
Venezia, A.M.
Venugopal, Jayarama R.
Veres, Teodor
Videira, Mafalda
Vilas, Jose Luis
Vosch, Tom
Wågberg, Thomas
Wang, Chenyu
Wang, Erkang
Wang, Huanting
Wang, Kuan-Wen
Wang, Pen-Cheng
Wang, Qiantao
Wang, Qin
Wang, Tanyu
Wang, Xiao
Wang, Xinghui
Wang, Yan Mei
Wang, Yun
Wang, Yunxiao
Weber, Stephen G.
Wei, Gang
Wei, Liping
Wei, Zhishun
Wen, Dan
Wheeler, Virginia
White, Jason
Windbergs, Maike
Witting, Paul
Wong, Danny K. Y.
Woo, Sungwook
Wu, Joseph
Wu, Si
Wujcik, Evan
Xi, Weixian
Xia, Tian
Xia, Weidong
Xiao, Xiaoyin
Xin, Guoqing
Xu, Leyuan
Yager, Paul
Yamamoto, Go
Yamauchi, Noriko
Yamauchi, Yusuke
Yan, Chenglin
Yanai, Nobuhiro
Yang, Bai
Yang, Fuqian
Yang, Nianjun
Yang, Ruidong
Yang, Shixuan
Yao, Jian
Yeo, Woon-Seok
Yeom, Guen Young
Yi, Dong Kee
Yoon, D. Y.
Yu, Hongtao
Yu, Meihua
Yu, Taekyung
Yu, Weijie
Yuan, Guanghui
Yuichi, Togashi
Yun, Yeoung-Sang
Zaraska, Leszek
Zhang, Chuanfang
Zhang, Longhe
Zhang, Qichun
Zhang, Sufeng
Zhang, Xiaoming
Zhang, Yixuan
Zhang, Yiying
Zhang, Z. L.
Zhou, Haoshen
Zhou, Nan
Zhu, Changbao
Zinchenko, Anatoly

